# Transforming health sciences library spaces

**DOI:** 10.29173/jchla29672

**Published:** 2023-04-01

**Authors:** Heather Cunningham

**Affiliations:** Assistant Director, Research & Innovation Services Gerstein Science Information Centre, University of Toronto, Toronto, ON, Canada

Campbell A. editor. **Transforming health sciences library spaces**. Lanham, MD: Rowman & Littlefield; 2019. Hardcover: 306 p. ISBN: 9781538114674. Price USD $94.00. Available from: https://rowman.com/ISBN/9781538114674/Transforming-Health-Sciences-Library-Spaces

The pressure on library spaces has been increasing over the past three decades from a variety of sources: changing institutional priorities, patron behaviours and expectations, collection development practices compounded with shrinking budgets, and evolving services and resources. Conversations around physical space combined with the library’s development as a welcoming and engaging virtual space or hub contribute to “library as place” being a robust and vigorous topic of discussion. Transforming health sciences library spaces adds ten case studies to this conversation. The chapters delve into the experiences of academic, hospital, and consumer health libraries that serve populations of students, faculty, health care providers, and patients. They focus predominantly on libraries in the United States but include one case study from the Health Sciences Library at the Northern Ontario School of Medicine (NOSM) University in Northern Ontario. With the exception of some of the hospital structures and funding models, the experiences and methodologies are transferable to the Canadian context. The writing quality is clear but at times somewhat inconsistent given a variety of authors. As described in the preface, these chapters “provide insights into planning, budgeting, collecting and integrating user feedback, collaborating with leadership and architects, and thriving in the good times and the tight times”.

This book is edited by Alanna Campbell, Public Services Librarian at the NOSM University Health Sciences Library. Her research interests include library spaces and user engagement. She has conducted research in these areas, presented at conferences, and co-authored articles on library spaces. Campbell also co-authored the last chapter in this book about practical advice and steps on how to plan and gradually implement space improvements within a library’s operational budget.

This work is divided into three sections: (i) library spaces that work for users; (ii) working in unique spaces; and (iii) library spaces working with what they’ve got. Common themes across the four case studies of the first section relate to the critical importance of understanding patron needs and the spaces and services required to address these needs, involving patron input in designing spaces, assessing how current spaces are used and why, and aligning with institutional strategic initiatives. Roksandic and Erlinger’s chapter called Consumer health library spaces discusses the challenges of providing consumer health information and services in a multi-site environment and concludes there was more engagement when these services were offered in community environments rather than library spaces. Their key takeaway is to carefully consider the role and balance of the library as an information provider and as a community space. Roksandic and Erlinger coauthor a second chapter in this section about the successful transformation of Mount Carmel Health System Library with several inspiring before and after photos. The marketing and communication of reenergized spaces was integral to this project as was listening to patrons to create much needed prayer and lactation rooms. A key takeaway is that physical and virtual spaces can no longer be considered in isolation – they are two facets of the same library. The Lackey et al. chapter on the methodology used to create a snapshot of library usage through a mixed methods design based on Nancy Fried Foster’s seminal ethnographic research (and engaging Foster as the project’s consultant) will be particularly interesting for those looking to replicate space usage research. Libraries transforming from collection based spaces into digital maker space hubs is the focus of the Minson et al. case study at the Marston Science Library at the University of Florida. Key takeaway messages emphasized the importance of planning, campus partnerships and champions, and communicating your successes.

The second section has a strong leadership theme. The Blackwell chapter on transforming a Chamberlain University library from a campus based space to a primarily virtual offering has valuable lessons pertaining to staffing, services, and enterprise software and infrastructure all the while grappling with questions around user’s changing experiences and perception of the library if it is primarily online. The Epstein chapter describes leadership, with examples of self leadership as a solo librarian, however the connections to the virtual library spaces were quite tenuous. The Carrigan and Burford chapter about the Medical Sciences Library at Texas A&M University was extremely interesting and well written. Their case study was a proactive strategic decision to renovate their physical space to emphasize a special collection and energize its relationship with space and patrons. With the growing homogeneity amongst library collections due to online resource packages, special or unique collections provide strength as “an effective tool in campus development, fund-raising, and alumni relations”. Their before and after space renovation description chart was very informative.

The final section emphasizes practicality. Decaro and Butcheck, of the Health Center Library at Case Western Reserve University, describe in detail the steps and planning they quickly had to undertake upon receiving the sudden directive the library was to lose half its space. A strength of this case study is the honesty of the authors in revealing the time consuming and workload-increasing mistakes that were made, so that others can learn from them and avoid similar issues. The Hoogland chapter is interesting as the authors show how being freed from the circulation desk and physical spaces in general, has facilitated the development of new roles in librarianship, such as research data management, institutional repository management, scholarly communications, liaison, assessment, and health and wellness. This chapter is useful for those new to librarianship. The final chapter in this work, Campbell and Fink’s case study on how libraries can improve spaces, signage, furniture, and add new technology with no funding or budget increase, is the most pragmatic. A clear vision and careful long-term planning can maximize any wiggle-room in one’s operating budget to implement incremental improvements to both user and staff spaces. This chapter is full of clear examples, recommended resources, as well as planning tips.

The ongoing COVID pandemic has forced us to reevaluate our relationship with, and usage of, physical and virtual spaces. Large societal and cultural changes over the past several years have made us be more conscious of how inclusive and accessible physical and virtual spaces need to be and what messages they relay about who is welcome to use them. Work is moving forward to decolonize library spaces in academic, public, and hospital libraries. The lenses of diversity, equity, decolonization, and accessibility with which we look at spaces now, however, are not strongly reflected in this 2019 book which the title words of “transforming spaces” may evoke. However, many important themes of collaborating with users, leadership, and campus partners, proactively assessing collection space requirements compared to space needs by patrons, conducting usability tests on virtual spaces, communicating your successes, and demonstrating the library’s relevance to its institution are timeless. This book would make an important addition to a collection on library as space because of its focus on health science libraries and especially if combined with the seminal works of Library as place: history, community and culture edited by Buschman and Leckie, and Library as place: rethinking roles, rethinking space edited by Freeman. A search of the recent journal or conference literature will find numerous articles on decolonizing spaces and making spaces (virtual and physical) welcoming and usable for diverse users.
